# Recently reported SARS-CoV-2 genomes suggested to be intermediate between the two early main lineages are instead likely derived

**DOI:** 10.1093/ve/veaf008

**Published:** 2025-02-22

**Authors:** Jonathan E Pekar, Niema Moshiri, Philippe Lemey, Alexander Crits-Christoph, Florence Débarre, Stephen A Goldstein, Zach Hensel, Andrew Rambaut, Michael Worobey, Edward C Holmes, Joel O Wertheim

**Affiliations:** Institute of Ecology and Evolution, University of Edinburgh, Ashworth Laboratories, Charlotte Auerbach Rd, Edinburgh EH9 3FL, United Kingdom; Department of Medicine, University of California San Diego, 9500 Gilman Drive, La Jolla, CA 92093, United States; Department of Computer Science & Engineering, University of California San Diego, 9500 Gilman Drive, La Jolla, CA 92093, United States; Department of Microbiology, Immunology and Transplantation, Rega Institute, Laboratory for Clinical and Epidemiological Virology, KU Leuven, Herestraat 49, Leuven BE-3000, Belgium; Independent Researcher; Institut d’Écologie et des Sciences de l’Environnement (IEES-Paris, UMR 7618), CNRS, Sorbonne Université, UPEC, IRD, INRAE, Paris, France; Department of Human Genetics, University of Utah School of Medicine, Salt Lake City, UT 84112, United States; Howard Hughes Medical Institute, 4000 Jones Bridge Road Chevy Chase, MD 20815, United States; Instituto de Tecnologia Química e Biológica António Xavier, Universidade Nova de Lisboa, Av. da República, Oeiras 2780-157, Portugal; Institute of Ecology and Evolution, University of Edinburgh, Ashworth Laboratories, Charlotte Auerbach Rd, Edinburgh EH9 3FL, United Kingdom; Department of Ecology and Evolutionary Biology, University of Arizona, Tucson, AZ 85721, United States; School of Medical Sciences, The University of Sydney, Sydney, NSW 2006, Australia; Department of Medicine, University of California San Diego, 9500 Gilman Drive, La Jolla, CA 92093, United States

**Keywords:** virus evolution, SARS-CoV-2, molecular clock

## Abstract

Understanding the genomic diversity of severe acute respiratory syndrome coronavirus 2 (SARS-CoV-2) at the outset of the coronavirus disease 2019 pandemic can provide insight into the circumstances leading to its emergence. Early SARS-CoV-2 genomic diversity has been classified into two distinct viral lineages, denoted “A” and “B,” which we hypothesized were separately introduced into humans. Recently published data contain two genomes with a haplotype suggested to be an evolutionary intermediate to these two lineages, known as “T/T.” We used a phylodynamic approach to analyze SARS-CoV-2 genomes from early 2020 to determine whether these two T/T genomes represent an evolutionarily intermediate haplotype between lineages A and B, or if they are a later descendent of either of these two lineages. We find that these two recently published T/T genomes do not represent an evolutionarily intermediate haplotype and were, instead, derived from either lineage A or lineage B. However, we cannot conclusively determine from which lineage they were derived. After including additional data from the start of the pandemic, including these two T/T genomes, we again find a discrepancy in the molecular clock when inferring the ancestral haplotype of SARS-CoV-2, corroborating existing evidence for the separate introductions of SARS-CoV-2 lineages A and B into the human population in late 2019.

## Introduction

The early part of the coronavirus disease 2019 (COVID-19) pandemic was characterized by the presence of two lineages, denoted A and B, that differed by two nucleotide substitutions (C8782T and T28144C, relative to lineage B, giving lineage A a “T/C” pattern and lineage B a “C/T” pattern at those key sites) (Rambaut et al. 2020). The Huanan Seafood Wholesale Market in Wuhan, China, was the early epicenter of the COVID-19 pandemic (Worobey et al. 2022), and these two lineages appear to have been separate introductions at the market (Pekar et al. 2022) based on molecular clock dating, the apparent lack of evolutionarily intermediate haplotypes, the geographic proximity of both lineages to the market (Worobey et al. 2022), and the sampling of genomes from both lineages at the market (Liu et al. 2024).

The discovery of a virus in humans from early in the pandemic with a haplotype intermediate to lineages A and B—possessing either C8782 and C28144 (i.e. a C/C haplotype) or T8782 and T28144 (i.e. a T/T haplotype)—could suggest that such an intermediate haplotype is an evolutionary intermediate between lineages A and B. In turn, such an evolutionary intermediate haplotype could indicate that lineages A and B were the result of a single introduction, where lineage A evolved from lineage B (or vice versa) in humans. Up until the publication of Lv et al. (2024), available genomes with a T/T or C/C haplotype at the outset of the pandemic were determined to be likely artifacts of contamination or bioinformatics errors, rather than intermediates, based on these genomes sharing rare mutations with lineage A or lineage B genomes, having poor sequencing depth at 8782 or 28144, or having an indeterminate nucleotide at 8782 (Pekar et al. 2022). However, the T/T haplotype has repeatedly arisen later throughout the pandemic, representing a derived (i.e. not transitional) haplotype and, accordingly, found with additional substitutions (Pekar et al. 2022). Such haplotypes emerge whenever a C-to-T mutation occurs at either site 8782 in a lineage A virus or site 28144 in a lineage B virus. Due to the C-to-T mutational bias of severe acute respiratory syndrome coronavirus 2 (SARS-CoV-2) in humans (De Maio et al. 2021, Pekar et al. 2022), derived T/T haplotypes are more commonly observed than derived C/C haplotypes (Chen et al. 2022).

In 2024, SARS-CoV-2 sequences from samples collected from 267 patients admitted to the Shanghai Public Health Clinical Center between January and September 2020 in Shanghai, China, resulting in complete genomes from 74 patients from January and February, were published (Lv et al. 2024). Two of these genomes, from patients sampled on 8 February and 11 February, were unambiguously assigned as T/T and did not possess any additional substitutions. Based on a maximum likelihood inference that does not incorporate sample dates, Lv et al. (2024) concluded that these T/T haplotypes were evolutionary intermediates between lineages A and B. However, considering that these two genomes were sampled months after the emergence of SARS-CoV-2, allowing additional time for both recurrent and unique substitutions, it is crucial to account for when these viruses were sampled when determining if they are evolutionary intermediates, rather than derived haplotypes. Here, we perform phylodynamic inference on genomes from early in the pandemic to determine whether these two genomes are likely evolutionary intermediates to lineages A and B.

## Methods

### Dataset

To perform phylodynamic inference, we used the dataset of 863 SARS-CoV-2 genomes from Crits-Christoph et al. (2024), which is a combination of the sequence data from Pekar et al. (2022), Liu et al. (2024), and Lv et al. (2024). Briefly, this dataset included 4 genomes isolated from environmental samples in the Huanan Seafood Wholesale Market in Wuhan (Liu et al. 2024), 785 SARS-CoV-2 genomes from Pekar et al. (2022), and 74 SARS-CoV-2 genomes from Lv et al. (2024). Among the 400 genomes available in Lv et al. (2024), we filtered genomes collected by 14 February 2020 and then used only the earliest sampled complete genome with <5% ambiguous nucleotides from each patient. Further details on the reconstruction of genomes isolated from environmental samples are provided in Crits-Christoph et al. (2024). There were a total of 863 SARS-CoV-2 genomes, each sampled by 14 February 2020. These genomes were already aligned using Multiple Alignment using Fast Fourier Transform (Katoh and Standley 2013), with Wuhan Hu-1 (accession number NC_045512) as a reference genome. In the dataset, there are three genomes with an ‘N’ at site 8782 (accession numbers OR240400, OR240405, and OR240361). Based on shared substitutions with lineage A (defined by 8782T and 28144C) and lineage B (defined by 8782C and 28144T) genomes, OR240400 can be confidently resolved as lineage A and OR240405 can be confidently resolved as lineage B. The genome OR240361 possesses a substitution (C3768T) present in both lineage A and lineage B genomes in our dataset and its haplotype is therefore unknown. We therefore have 279 lineage A genomes, 581 lineage B genomes, two T/T genomes, and one genome with an unresolved haplotype in our dataset. The accession IDs for all the genomes can be found in Supplementary Tables S4 (all) and S5 (GISAID).

### Phylogenetic inference

We performed Bayesian molecular clock phylodynamic inference using BEAST v1.10.5 (Suchard et al. 2018) following the same protocol as in Pekar et al. (2022), employing a nonreversible, random-effects substitution model (Magee et al. 2024), a strict molecular clock, and a nonparametric skygrid prior (Gill et al. 2013) with 20 regular grid points and the last point at 0.37, which corresponds to 5 October 2019. As in Pekar et al. (2022), we performed an unconstrained analysis using just the 863 SARS-CoV-2 genomes and another analysis constrained using the recombinant common ancestor (recCA) of SARS-CoV-2. The recCA is an inferred ancestral sequence of SARS-CoV-2 based on the phylogenetic reconstruction of sarbecoviruses closely related to SARS-CoV-2 while accounting for recombination [the recCA sequence was identical to the one from Pekar et al. (2022)]. For both analyses, we ran four independent chains of 500 million generations, subsampling every 50 thousand iterations to continuous parameter log files, every 200 thousand iterations to continuous ancestral state reconstruction log files, and every 200 thousand iterations to continuous tree log files. The first 10% of each chain was discarded as burn-in, convergence and mixing were assessed in Tracer v1.7.1(Rambaut et al. 2018), and the four chains for a given analysis were combined using LogCombiner. All relevant effective sample size values were >150 for the combined log file for each analysis.

For sensitivity analyses, we repeated the recCA-constrained inference using (i) site-to-site rate variation with eight equally weighted Γ categories, (ii) an uninformative continuous-time Markov chain molecular clock prior, and (iii) downsampling of lineage A or lineage B genomes. For (iii), we performed four separate inferences: downsampling lineage A genomes by 50% or 75%, or downsampling lineage B genomes by 50% or 75% (we refer to the approximate relative proportions of lineage B to lineage A genomes in Fig. 2 as the “B:A ratio”).

We then repeated the recCA-constrained inference while simultaneously inferring the sampling dates of the two T/T genomes, given a uniform prior ranging from 14 February 2019 to 14 February 2020. We additionally performed recCA-constrained inferences while specifying earlier sampling dates for the two T/T genomes, ranging from the start of January 2020 to their true sampling dates in February 2020 (Fig. 3).

We next determined whether either of the two genomes in the Lv et al. (2024) dataset with 8782T and 28144T (henceforth referred to as T/T genomes) were evolutionary intermediates to lineage A and lineage B for each posterior tree. If the ancestral haplotype was lineage A, a T/T genome would be considered an evolutionary intermediate if it diverged from the branch leading to lineage B. Similarly, if the ancestral haplotype was lineage B, a T/T genome would be considered an evolutionary intermediate if it diverged from the branch leading to lineage A. If the ancestral haplotype was T/T, then a T/T genome would be considered an evolutionary intermediate if it diverged from the branch leading to lineage A or lineage B (i.e. the branch connecting those two lineages). In other words, if a T/T genome was basal to lineage A or lineage B, and not a descendant of either lineage, it would be intermediate to the two of them. If the ancestral haplotype was C/C, a T/T genome would be considered an evolutionary intermediate if it emerged on the branch separating lineage A and lineage B. Note that this latter scenario would imply there were both the C8782T and C28144T substitutions leading to the T/T genome(s), and then additional substitution at one of these two sites along the branch to lineage A or lineage B.

We calculated Bayes factor (BF) support based on the approach in Pekar et al. (2022). Briefly, the BF supporting the rejection of a given ancestral haplotype is calculated by dividing the posterior probability of the majority ancestral haplotype by the posterior probability of the given ancestral haplotype. Note that, unlike in our previous analysis, we pooled all ancestral haplotypes with additional derived substitutions into the given ancestral haplotype (e.g. all ancestral haplotypes corresponding to lineage A plus additional substitutions are pooled into what we call lineage A). All inferred ancestral haplotypes for the unconstrained and recCA-constrained analyses can be found in Supplementary Tables S1 and S2, respectively. The lineage A and lineage B ancestral haplotypes with derived mutations are collapsed into lineage A and lineage B, respectively, in Tables 1 and 2.

**Table 1.** Posterior frequencies of monophyly and lineage placement of T/T genomes when performing an unconstrained inference, stratified by inferred ancestral haplotype.

Ancestral haplotype	Total	Monophyletic	Derived from A	Derived from B	Derived from A and B	Evolutionary intermediate
A (T/C)	737	734 (99.6%)	260 (35.3%)	476 (64.6%)	1 (0.1%)	0 (0.0%)
B (C/T)	5390	5367 (99.6%)	1817 (33.7%)	3546 (65.8%)	7 (0.1%)	20 (0.4%)
C/C	2655	2643 (99.5%)	867 (32.7%)	1782 (67.1%)	6 (0.2%)	0 (0.0%)
T/T	218	216 (99.1%)	76 (34.9%)	140 (64.2%)	1 (0.5%)	1 (0.5%)
Total	9000	8960 (99.6%)	3020 (33.6%)	5944 (66.0%)	15 (0.2%)	21 (0.2%)

Refer to Supplementary Table S1 for ancestral haplotypes expanded to showcase derived substitutions.

**Table 2.** Posterior frequencies of monophyly and lineage placement of T/T genomes when performing a recCA-constrained inference, stratified by inferred ancestral haplotype.

Ancestral haplotype	Total	Monophyletic	Derived from A	Derived from B	Derived from A and B	Evolutionary intermediate
A (T/C)	8216	8140 (99.1%)	2834 (34.5%)	5348 (65.1%)	26 (0.3%)	8 (0.1%)
B (C/T)	83	83 (100.0%)	26 (31.3%)	57 (68.7%)	0 (0.0%)	0 (0.0%)
C/C	539	535 (99.3%)	191 (35.4%)	347 (64.4%)	1 (0.2%)	0 (0.0%)
T/T	162	160 (98.8%)	59 (36.4%)	102 (63.0%)	0 (0.0%)	1 (0.6%)
Total	9000	8918 (99.1%)	3110 (34.6%)	5854 (65.0%)	27 (0.3%)	9 (0.1%)

Refer to Supplementary Table S2 for ancestral haplotypes expanded to showcase derived substitutions.

## Results

**Figure 1.**
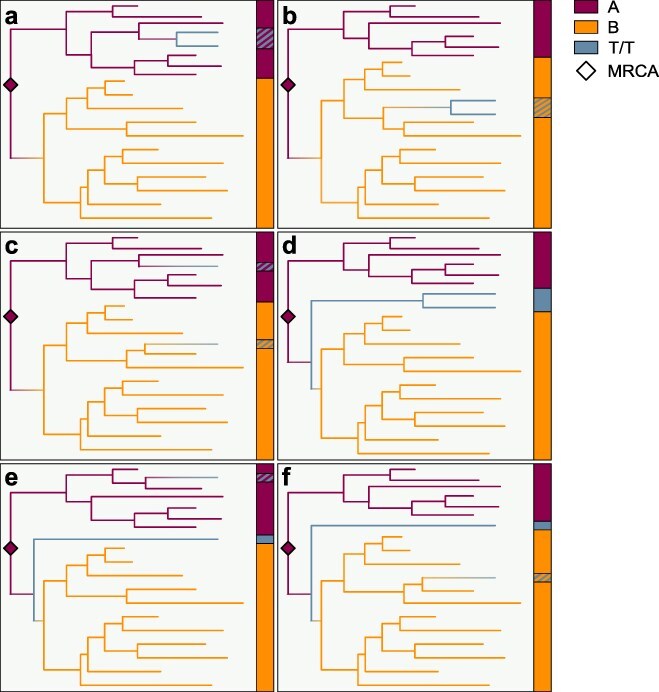
Schematic of time-scaled phylogenies depicting inferred lineage A ancestral haplotype and T/T lineage placement. Scenarios in which the two T/T genomes are (a) derived from lineage A, (b) derived from lineage B, (c) separately derived from lineages A and B and neither are intermediates, (d) intermediate genomes arising along the evolution to lineage B, (e) composed of one intermediate genome arising along the evolution to lineage B and one genome derived from lineage A, and (f) composed of one intermediate genome arising along the evolution to lineage B and one genome derived from lineage B. Horizontal axis is time. Colors of the vertical bars indicate the lineage of sampled taxa; colored hashes over the blue indicate what haplotypes the T/T genomes were derived from. No hash is present when the T/T genomes are inferred to be intermediate genomes. Refer to Supplementary Figs S1 and S2 for schematics with lineage B and T/T ancestral haplotypes, respectively.

The two T/T genomes found in Shanghai, SH-P37-2 and SH-P55-2, do not share any substitutions found within lineage A or lineage B that could indicate whether these genomes descend from either lineage (Worobey et al. 2020, Pekar et al. 2022), thereby making phylodynamic inference necessary to determine their phylogenetic placement. To determine whether the two T/T genomes are evolutionary intermediate genomes, or are derived from either lineage A or B, we performed Bayesian molecular clock phylodynamic inference on a dataset comprising SARS-CoV-2 genomes representing the global diversity of SARS-CoV-2 at the start of the pandemic (*n* = 863 genomes collected by 14 February 2020; see Methods). We inferred the ancestral haplotype of the most recent common ancestor (MRCA) of the SARS-CoV-2 genomes as well as the phylogenetic position of each of the T/T genomes: (i) derived from lineage A, (ii) derived from lineage B, or (iii) an evolutionary intermediate where the T/T genome is descended from the branch connecting lineage A and lineage B (Fig. 1, Supplementary Figs S1–S2). We compared these three scenarios using posterior probabilities, the frequencies at which different evolutionary histories were sampled during Bayesian phylodynamic inference.

We found that the two T/T genomes from Shanghai were rarely inferred to be evolutionary intermediates in our phylodynamic analysis (posterior probability that they are evolutionary intermediates, *P* = .002), regardless of the inferred ancestral haplotype (Table 1). Even when the inferred ancestral haplotype is T/T (as in 2.4% of sampled topologies; Table 1, calculated based on the “Total” column), these two T/T genomes were still determined to be derived from lineage A or lineage B (*P* = .995). Whether the T/T genomes are derived from lineage A or lineage B is uncertain. The posterior probability of the T/T genomes being derived from lineage A (*P* = .336) or lineage B (*P* = .660) is proportional to the relative number of lineage A (*n* = 279/860; 32.4%) and lineage B (*n* = 581/860; 67.6%) genomes in the dataset (see Methods). The two T/T genomes published by Lv et al. (2024) appear monophyletic (*P* = .996).

**Figure 2.**
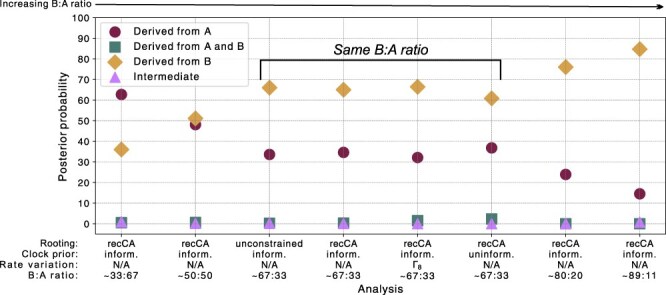
Posterior probability of the T/T genomes being either an evolutionary intermediate or derived. Rooting indicates whether the analysis used unconstrained rooting or recCA-rooting. Clock prior indicates whether the analysis used an informative (i.e. based on a previous study) or uninformative (continuous-time Markov chain) prior for the evolutionary rate. Rate variation denotes whether the substitution model incorporated site-to-site rate variation. The B:A ratio indicates the approximate ratio of lineage B to lineage A genomes (see Methods). The relative proportion of lineage B to lineage A genomes increases from left to right along the *x*-axis. Note that the posterior probability values are the sums across all ancestral haplotypes and are therefore equivalent to the “Total” row in Tables 1–3 for each analysis.

Next, we repeated a phylodynamic inference of the same dataset, this time constraining the rooting of the topology with the recCA: an ancestral sequence inferred using SARS-CoV-2 and closely related bat sarbecoviruses while accounting for recombination (Pekar et al. 2022). Our results were consistent with the unconstrained analysis, with T/T genomes again rarely inferred to be true intermediates (*P* = .001; Fig. 1). These genomes again appear monophyletic (*P* = .991), and their probability of being derived from lineage A or lineage B was concordant with the proportion of lineage A and lineage B genomes in the dataset (Table 2). Consistent results were obtained when introducing site-to-site variation in our inference (Γ_8_) or using an uninformative molecular clock prior (Fig. 2), and the posterior probabilities of being derived from lineage A or lineage B were again concordant with the proportion of lineage A and B genomes in the dataset.

We next tested whether the frequency with which T/T genomes are derived from either lineage A or lineage B was related to the proportion of lineage A or lineage B genomes in our dataset by separately downsampling lineages A and B. This approach revealed that the frequency with which T/T genomes were derived from lineage A increased when downsampling lineage B, and, conversely, decreased when downsampling lineage A (Fig. 2). Hence, although our results indicate that these T/T genomes are very likely derived, we cannot conclusively determine from which lineage they arose.

The ancestral haplotype when using an unconstrained rooting is uncertain, although it was more likely lineage B (59.9% of sampled topologies), but we can confidently reject the T/T ancestral haplotype (BF = 24.7). When rooting with the recCA, the most likely ancestral haplotype was lineage A (91.3% of sampled topologies; Table 2), and we can reject all the other ancestral haplotypes, including lineage B (BF = 15.2). Even after including more data than in our previous analyses (Pekar et al. 2022), we found that a lineage A ancestral haplotype was more likely with the recCA-constrained inference and that a lineage B ancestral haplotype was more likely with an unconstrained inference.

**Figure 3.**
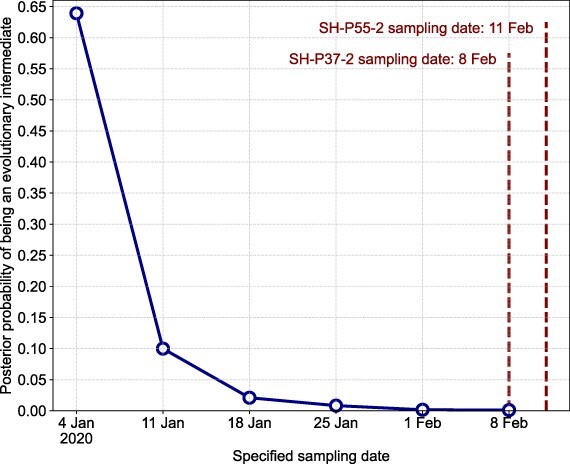
Probability of an evolutionary intermediate existing when specifying earlier T/T sampling dates. Posterior probability of at least one of the two genomes being an evolutionary intermediate when specifying artificially earlier sampling dates for the two T/T genomes. The date shown at each time point is the specified date of sampling of SH-P37-2; the sampling date of SH-P55-2 is always 3 days later, as is the real date. The true sampling dates of SH-P37-2 and SH-P55-2 are 8 February 2020 and 11 February 2020, respectively.

To determine when a true evolutionary intermediate with a T/T haplotype could have existed in humans, we next performed phylogenetic inference while assuming unknown sampling dates for the T/T genomes. We found that the theoretical T/T genomes with indeterminate sampling dates were consistently inferred to be evolutionary intermediates (*P* > .999; Table 3) and increased the support for a T/T ancestral haplotype (46.7% of sampled topologies). However, the inferred sampling dates of these theoretical intermediate T/T genomes precede 24 December 2019, the date of the earliest sampled SARS-CoV-2 genome, with both T/T genomes having an inferred sampling date of 7 December 2020 (95% highest posterior density: 11 November 2020–23 December 2020). These results demonstrate that the date of sampling of these two genomes is one key characteristic that influences whether they would be inferred as evolutionary intermediates. We therefore performed additional phylogenetic inference while specifying different counterfactual sampling dates for the two T/T genomes, ranging from the start of January 2020 to their true sampling dates in February 2020. This analysis showed that had these two T/T genomes been sampled at the start of January 2020, they could not be rejected as evolutionary intermediates (*P* = .639). However, as the specified sampling dates progress toward the end of January 2020, it becomes increasingly unlikely that T/T genomes would represent evolutionary intermediates (Fig. 3).

**Table 3.** Posterior frequencies of monophyly and lineage placement of T/T genomes when simultaneously performing a recCA-constrained inference of tree topology and the sampling dates of the T/T genomes (assumed to be unknown), stratified by inferred ancestral haplotype.

Ancestral haplotype	Total	Monophyletic	Derived from A	Derived from B	Derived from A and B	Evolutionary intermediate
A (T/C)	4772	1507 (31.6%)	1 (0.0%)	0 (0.0%)	0 (0.0%)	4771 (100.0%)
B (C/T)	23	8 (34.8%)	0 (0.0%)	0 (0.0%)	0 (0.0%)	23 (100.0%)
C/C	0	N/A	N/A	N/A	N/A	N/A
T/T	4205	581 (13.8%)	0 (0.0%)	0 (0.0%)	0 (0.0%)	4205 (100.0%)
Total	9000	2096 (23.3%)	1 (0.0%)	0 (0.0%)	0 (0.0%)	8999 (100.0%)

Refer to Supplementary Table S3 for ancestral haplotypes expanded to showcase derived substitutions.

## Discussion

Our analysis demonstrates that the two SARS-CoV-2 T/T genomes collected in Shanghai in February 2020 by Lv et al. (2024) are extremely unlikely to represent evolutionary intermediates between lineage A and lineage B. Because of their relatively late sampling dates, as well as the C-to-T mutational bias that characterizes SARS-CoV-2, these T/T genomes were most probably derived from one of the two founding lineages, although we were unable to conclusively determine which lineage they arose from.

The conclusion that lineage B emerged first—despite lineage A being more closely related to bat coronaviruses (Pekar et al. 2022)—combined with the geographic proximity of lineage A and lineage B cases to the Huanan market (Worobey et al. 2022) and the detection of virus from both lineages at the Huanan market (Crits-Christoph et al. 2024), point toward the separate zoonotic introductions of lineage A and lineage B at the Huanan market (Liu et al. 2024). That these two T/T genomes are derived, rather than intermediate, means that there is still no evidence that either lineage A or lineage B evolved from their counterpart in humans.

Considering the C-to-T mutational bias of SARS-CoV-2 (De Maio et al. 2021, Pekar et al. 2022), genomes with an evolutionarily intermediate T/T haplotype would have had to have appeared substantially earlier in the pandemic than the ones sampled by Lv et al. (2024) (i.e. likely before mid-January 2020, see Fig. 3) to be suggestive of lineage B evolving from lineage A (or vice versa) in humans; however, low rates of sequencing early in the pandemic could have prevented the evolutionary intermediates from being identified. Nonetheless, considering the time of the MRCA of lineages A and B in 2019 (Pekar et al. 2022), it is unlikely that a true evolutionary intermediate would have accrued no additional mutations by February 2020. Therefore, if the transition between lineages A and B occurred in the human population and the evolutionary intermediates had survived until mid-February 2020, we would have likely observed their evolutionary descendants, even if we failed to observe the initial intermediates themselves.

Although our analyses indicate that the two T/T genomes shared by Lv et al. (2024) are likely derived, our analyses do not preclude the possibility that actual but unobserved evolutionary intermediates existed in humans: if both lineages A and B evolved in animals, evolutionary intermediate genomes could have spilled over at the Huanan market (Pekar et al. 2022), but their lineage went extinct.

Like the T/T genomes that arose during the Diamond Princess outbreak in early 2020 (Sekizuka et al. 2020, Murata et al. 2021), the T/T genomes from Shanghai appear to be monophyletic. Although they could represent a single transmission chain, one of the two patients was infected in Henan province, China, whereas the other was infected in Shanghai and was in contact with an individual who had been infected in Wuhan. If these infections were not the result of a single transmission chain, they could indicate the repeated evolution of a T/T haplotype, as has been seen elsewhere.

The genomic data published by Lv et al. (2024) have provided additional clarity regarding the early pandemic. If available, the publication of additional genomic data from the start of the pandemic could shed further light on the emergence, evolution, and spread of SARS-CoV-2.

## Supplementary Material

veaf008_Supp

## Data Availability

XML and codes are available at https://github.com/pekarj/SC2_intermediates.
