# Supplementary material for: Recently reported SARS-CoV-2 genomes suggested to be intermediate between the two early main lineages are instead likely derived
Source: Virus Evol. 2025 Feb 22;11(1):veaf008. doi: 10.1093/ve/veaf008 (PMC11878783; doi:10.1093/ve/veaf008)
Supplement: veaf008_Supp [file veaf008_supp.zip › suppl_data/Supplementary_Information.docx]

**Table S1.** Posterior frequencies of ancestral haplotypes including derived substitutions when performing the unconstrained inference.

| **Haplotype** | **Mutations from Hu-1 reference** | **Count** | **Proportion** |
| --- | --- | --- | --- |
| B (C/T) | N/A | 5145 | 0.571667 |
| C/C | T28144C | 2654 | 0.294889 |
| A (C/T) | T28144C+C8782T | 723 | 0.080333 |
| T/T | C8782T | 218 | 0.024222 |
| B+C6968A (C/T) | C6968A | 96 | 0.010667 |
| B+T11764A (C/T) | T11764A | 45 | 0.005 |
| B+C6968A+T11764A (C/T) | C6968A+T11764A | 17 | 0.001889 |
| B+T27384C (C/T) | T27384C | 9 | 0.001 |
| B+T565C (C/T) | T565C | 9 | 0.001 |
| B+T565C+T27384C (C/T) | T565C+T27384C | 8 | 0.000889 |
| B+A24325G (C/T) | A24325G | 8 | 0.000889 |
| B+G20670A (C/T) | G20670A | 7 | 0.000778 |
| B+G28183A (C/T) | G28183A | 6 | 0.000667 |
| A+T26729C (C/T) | T28144C+C8782T+T26729C | 6 | 0.000667 |
| B+G1397A (C/T) | G1397A | 6 | 0.000667 |
| B+G20679A (C/T) | G20679A | 5 | 0.000556 |
| B+T565C+T27384C+G29573A (C/T) | T565C+T27384C+G29573A | 3 | 0.000333 |
| B+T28688C (C/T) | T28688C | 3 | 0.000333 |
| A+T26729C+G28077C (C/T) | T28144C+C8782T+T26729C+G28077C | 3 | 0.000333 |
| A+G28077C (C/T) | T28144C+C8782T+G28077C | 3 | 0.000333 |
| B+G20670A+G20679A (C/T) | G20670A+G20679A | 3 | 0.000333 |
| B+G1397A+T28688C (C/T) | G1397A+T28688C | 3 | 0.000333 |
| B+T27384C+G29573A (C/T) | T27384C+G29573A | 3 | 0.000333 |
| B+C26370T (C/T) | C26370T | 2 | 0.000222 |
| B+C27944T (C/T) | C27944T | 2 | 0.000222 |
| C/C+T8294C | T28144C+T8294C | 1 | 0.000111 |
| B+T565C+G29573A (C/T) | T565C+G29573A | 1 | 0.000111 |
| B+C28093T (C/T) | C28093T | 1 | 0.000111 |
| B+T565C+C17825T (C/T) | T565C+C17825T | 1 | 0.000111 |
| A+A8173T+T20852A+C21997A (C/T) | T28144C+C8782T+A8173T+T20852A+C21997A | 1 | 0.000111 |
| A+C4893T+T22807C+T27986C (C/T) | T28144C+C8782T+C4893T+T22807C+T27986C | 1 | 0.000111 |
| B+G29573A (C/T) | G29573A | 1 | 0.000111 |
| B+A28305C (C/T) | A28305C | 1 | 0.000111 |
| B+T13270C (C/T) | T13270C | 1 | 0.000111 |
| B+A21137G (C/T) | A21137G | 1 | 0.000111 |
| B+C17825T (C/T) | C17825T | 1 | 0.000111 |
| B+G26144T (C/T) | G26144T | 1 | 0.000111 |
| B+C17288T (C/T) | C17288T | 1 | 0.000111 |

**Table S2.** Posterior frequencies of ancestral haplotypes including derived substitutions when performing the recCA-constrained inference.

| **Haplotype** | **Mutations from Hu-1 reference** | **Count** | **Proportion** |
| --- | --- | --- | --- |
| A (C/T) | T28144C+C8782T | 8116 | 0.901778 |
| C/C | T28144C | 539 | 0.059889 |
| T/T | C8782T | 162 | 0.018 |
| B (C/T) | N/A | 83 | 0.009222 |
| A+C29095T (C/T) | T28144C+C8782T+C29095T | 59 | 0.006556 |
| A+C18060T (C/T) | T28144C+C8782T+C18060T | 26 | 0.002889 |
| A+C24023T (C/T) | T28144C+C8782T+C24023T | 10 | 0.001111 |
| A+T26729C (C/T) | T28144C+C8782T+T26729C | 3 | 0.000333 |
| A+G28077C (C/T) | T28144C+C8782T+G28077C | 1 | 0.000111 |
| A+T4946C (C/T) | T28144C+C8782T+T4946C | 1 | 0.000111 |

**Table S3.** Posterior frequencies of ancestral haplotypes including derived substitutions when simultaneously performing a recCA-constrained inference of tree topology and sampling dates of the T/T genomes.

| **Haplotype** | **Mutations from Hu-1 reference** | **Count** | **Proportion** |
| --- | --- | --- | --- |
| A (C/T) | T28144C+C8782T | 4752 | 0.528 |
| T/T | C8782T | 4205 | 0.467222 |
| B (C/T) | N/A | 23 | 0.002556 |
| A+C29095T (C/T) | T28144C+C8782T+C29095T | 10 | 0.001111 |
| A+C24023T (C/T) | T28144C+C8782T+C24023T | 5 | 0.000556 |
| A+C18060T (C/T) | T28144C+C8782T+C18060T | 3 | 0.000333 |
| A+T29029C (C/T) | T28144C+C8782T+T29029C | 1 | 0.000111 |
| A+T26729C (C/T) | T28144C+C8782T+T26729C | 1 | 0.000111 |

**
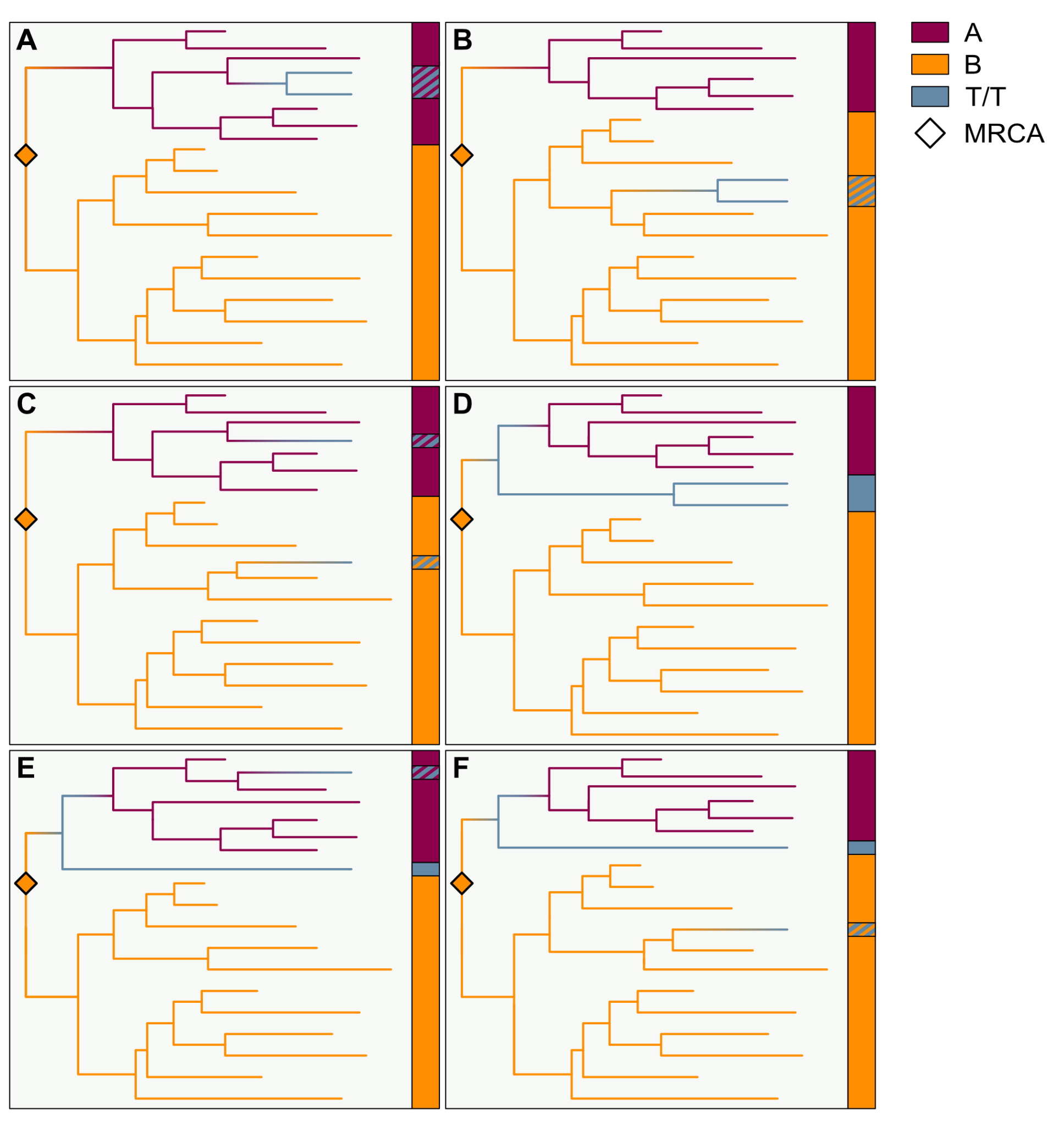
Figure S1.** **Schematic of time-scaled phylogenies depicting inferred lineage B ancestral haplotype and T/T lineage placement.** Scenarios in which the two T/T genomes are (A) derived from lineage A, (B) derived from lineage B, (C) separately derived from lineages A and B and neither are intermediates, (D) intermediate genomes arising along the evolution to lineage A, (E) composed of one intermediate genome arising along the evolution to lineage A and one genome derived from lineage A, and (F) composed of one intermediate genome arising along the evolution to lineage A and one genome derived from lineage B. Colors of the vertical bars indicate lineage of sampled taxa; colored hashes over the blue indicate what haplotypes the T/T genomes were derived from. No hash is present when the T/T genomes are inferred to be intermediate genomes. Refer to Figure 1 and Supplementary Figure S2 for schematics with lineage A and T/T ancestral haplotypes, respectively.

**
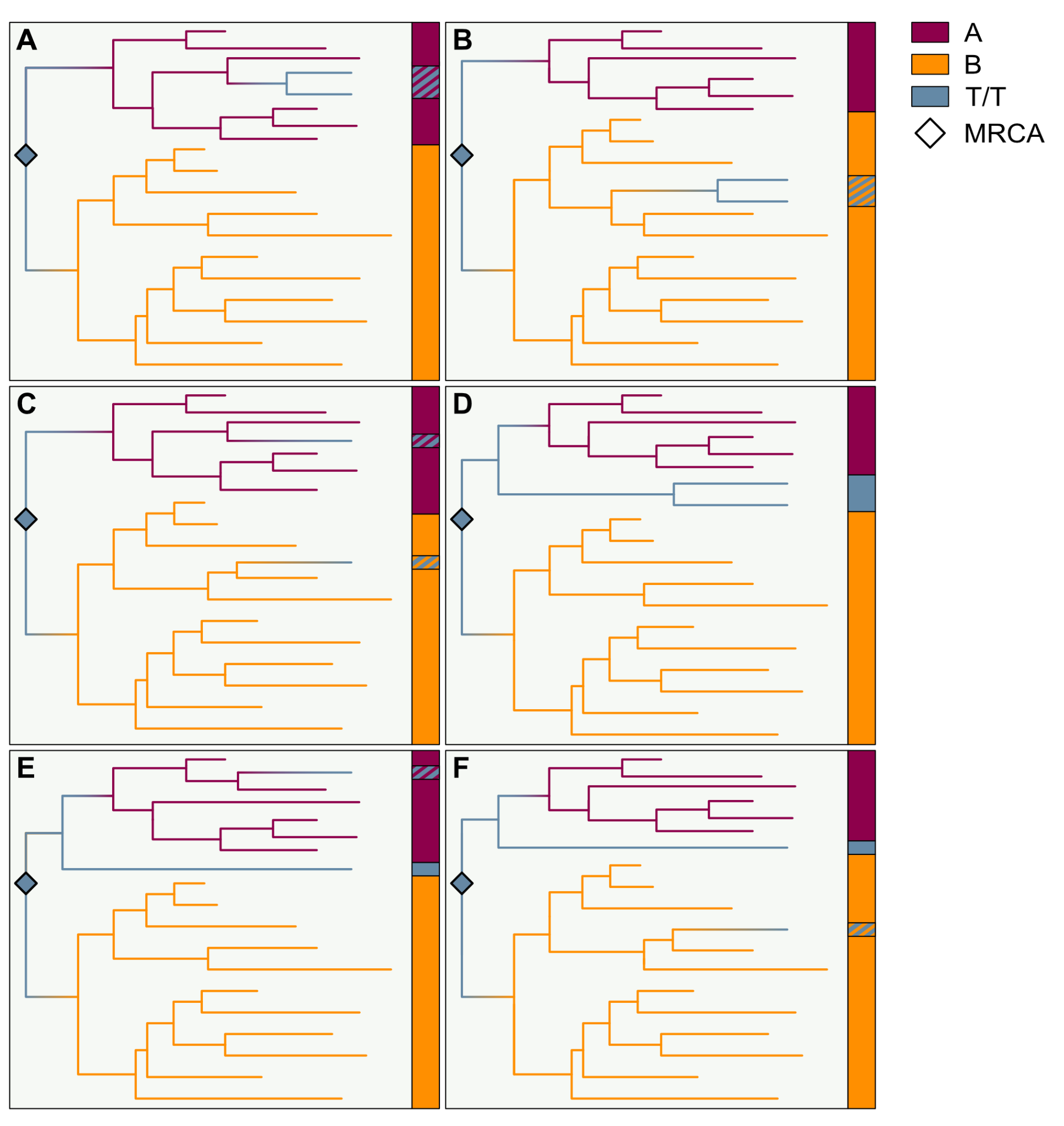
Figure S2.** **Schematic of time-scaled phylogenies depicting inferred T/T ancestral haplotype and T/T lineage placement.** Scenarios in which the two T/T genomes are (A) derived from lineage A, (B) derived from lineage B, (C) separately derived from lineages A and B and neither are intermediates, (D) intermediate genomes arising along the evolution to lineage A, (E) composed of one intermediate genome arising along the evolution to lineage A and one genome derived from lineage A, and (F) composed of one intermediate genome arising along the evolution to lineage A and one genome derived from lineage B. Note that (D,E,F) could, alternatively, have the evolutionary intermediate arise along the evolution to lineage B. Colors of the vertical bars indicate lineage of sampled taxa; colored hashes over the blue indicate what haplotypes the T/T genomes were derived from. No hash is present when the T/T genomes are inferred to be intermediate genomes. Refer to Figure 1 and Supplementary Figure S1 for schematics with lineage A and B ancestral haplotypes, respectively.
